# Co-Expression of Lipid Transporters Simultaneously Enhances Oil and Starch Accumulation in the Green Microalga *Chlamydomonas reinhardtii* under Nitrogen Starvation

**DOI:** 10.3390/metabo13010115

**Published:** 2023-01-10

**Authors:** Ru Chen, Yasuyo Yamaoka, Yanbin Feng, Zhanyou Chi, Song Xue, Fantao Kong

**Affiliations:** 1 School of Bioengineering, Dalian University of Technology, Dalian 116024, China; 2 Division of Biotechnology, Catholic University of Korea, Bucheon 420-743, Republic of Korea

**Keywords:** lipid transporters, oils, starch, triacylglycerol, nitrogen starvation, *Chlamydomonas reinhardtii*

## Abstract

Lipid transporters synergistically contribute to oil accumulation under normal conditions in microalgae; however, their effects on lipid metabolism under stress conditions are unknown. Here, we examined the effect of the co-expression of lipid transporters, fatty acid transporters, (FAX1 and FAX2) and ABC transporter (ABCA2) on lipid metabolism and physiological changes in the green microalga *Chlamydomonas* under nitrogen (N) starvation. The results showed that the TAG content in *FAX1-FAX2-ABCA2* over-expressor (OE) was 2.4-fold greater than in the parental line. Notably, in *FAX1-FAX2-ABCA2-OE*, the major membrane lipids and the starch and cellular biomass content also significantly increased compared with the control lines. Moreover, the expression levels of genes directly involved in TAG, fatty acid, and starch biosynthesis were upregulated. *FAX1-FAX2-ABCA2-OE* showed altered photosynthesis activity and increased ROS levels during nitrogen (N) deprivation. Our results indicated that *FAX1-FAX2-ABCA2* overexpression not only enhanced cellular lipids but also improved starch and biomass contents under N starvation through modulation of lipid and starch metabolism and changes in photosynthesis activity. The strategy developed here could also be applied to other microalgae to produce FA-derived energy-rich and value-added compounds.

## 1. Introduction

Microalgae are widely regarded as a promising sustainable source for biofuel and bioproduct production [1,2]. In nature, microalgae live in a dynamically changing environment. When they are subjected to stress conditions (e.g., nitrogen (N) starvation), nutrient assimilation is limited and growth is retarded [3]. The excess reducing power produced by photosynthesis may lead to photooxidative damage to the cells due to the generation of reactive oxygen species (ROS) [3,4]. Plants including microalgae assimilate and sequester the excess of carbon, electrons, and reducing equivalents as storage reserves in the form of neutral lipids (triacylglycerols, TAG) or high molecular weight carbohydrates (starch) to protect cells from oxidative damage [5,6]. Both TAG and starch are energy-rich compounds which can be converted to biodiesel and bioethanol, respectively [7]. 

In past decades, microalgae have been regarded as a promising renewable energy source to produce biofuels [8,9]. However, the content of TAG in microalgae is naturally low, which causes biofuel production from microalgae to be currently not economically viable [10,11]. In past decades, the key genes in TAG biosynthesis to enhance lipid production have been overexpressed, however, resulting in mixed success [11,12]. Therefore, more alternative strategies besides direct manipulation of the key enzymes in the TAG biosynthesis pathway to overproduce oils are required. 

Fatty acids (FA) are the primary building blocks for TAG biosynthesis in plants including microalgae. However, FA biosynthesis and incorporation of FA into TAG occur mainly within the chloroplast and the endoplasmic reticulum (ER), respectively [13,14]. During the consecutive FA transport from chloroplast to ER, FA exporters (FAX1 and FAX2) and ATP binding cassette (ABC) transporters of subfamily A (ABCA2) were found to play critical roles in the lipid metabolism of *Chlamydomonas* [15,16,17]. FAX1 is plastid-located, while FAX2 (also named Cr-FAX5) is targeted to ER. ABCA2 is an ER-located lipid transporter and is involved in lipid accumulation in *Chlamydomonas* [15]. The single over-expressor (FAX1- or ABCA2-OE) showed an increase in oil content [15,17]. However, in the strains with increased levels of *FAX2* only, the TAG content was not improved [17]. Whether the TAG accumulation in these single over-expressors is limited by the functional diversity or spatiotemporal expression regulation of these lipid transporters remains unknown. Previous studies have shown that N starvation can significantly induce the accumulation of lipids in microalgae [18,19]. Recently, we reported that co-expression of *FAX1-FAX2-ABCA2* significantly enhanced TAG accumulation in *Chlamydomonas* under normal growth conditions [20]. However, the effect of these lipid transporters on TAG and starch accumulation under N starvation and corresponding mechanisms have not yet been investigated. 

In this study, we examined the effect of the co-expression of lipid transporters (*FAX1*, *FAX2* and *ABCA2*) on TAG biosynthesis, lipid remodeling, and cellular responses in *Chlamydomonas* under N starvation. The results showed that *FAX1-FAX2-ABCA2* overexpression enhanced lipid accumulation, improved starch and biomass content, and caused changes in photosynthesis activity. The strategy developed here could be applied to other microalgae to produce FA-derived energy-rich and value-added compounds. 

## 2. Materials and Methods

### 2.1. Strains and Cultivation Conditions

The *Chlamydomonas reinhardtii* strain UVM4 [21] was used as the parental line for transgenetic line generation. The representative transgenic strains *FAX1-FAX2*-OE (*FAX1-FAX2-35*), *ABCA2*-OE (*ABCA2-2*), and *FAX1-FAX2-ABCA2*-OE (*FAX1-FAX2-ABCA2-51*) were generated in our previous studies [15,20]. The *Chlamydomonas* cells were cultivated in a tris-acetate phosphate (TAP) medium [22] with constant shaking (120 rpm) and continuous illumination (100 µmol m^−2^ s^−1^) at 25 °C in stackable incubator shakers (IS-6CL, Crystal Technology & Industries, Inc., Dallas, TX, USA). To induce nitrogen starvation (48 h), the cells initially grown in the TAP medium were washed and transferred to nitrogen-free TAP media (TAP-N). To analyze the cell number, *Chlamydomonas* cells were counted using an automated Algae Counter (Countstar BioMarine, Ruiyu Biotech Co., Shanghai, China). 

### 2.2. RNA Isolation and Quantification 

The total RNA was extracted using RNAiso Plus reagent (Takara Biomedical Technology (Beijing) Co., Beijing, China) following the manufacturer’s instructions. First-strand cDNA was synthesized using a SuperScript VILO cDNA Synthesis Kit (Thermo Fisher Scientific, Waltham, MA, USA) with 1.0 µg total RNA. Quantitative reverse transcription PCR (RT-qPCR) was performed on Applied Biosystems Quantstudio 5 using PowerUp SYBR green master mix (Thermo Fisher Scientific, Waltham, MA, USA). The 2^−ΔΔCT^ method [23] was used to analyze the gene expression, and the transcript levels were normalized to internal control *RACK1* (Cre06.g278222). The primer sequences used in this study are shown in Table S1.

### 2.3. Lipid Extraction and Quantification

The lipids were extracted using the modified Bligh and Dyer method as described previously [24,25]. To quantify the TAG, the extracted total lipids were separated on a thin-layer chromatography (TLC) plate using a solvent mixture consisting of acetic acid- diethyl ether-hexane (1:30:80, by volume). To quantify the membrane lipids, the extracted total lipids were separated on the TLC plate by a solvent mixture of water-acetic-acid-methanol-chloroform (3:9:13:75, by volume). The lipids were recovered from the TLC plate and transmethylated into fatty acid methyl esters (FAMEs) using the direct transmethylation method [20,24]. The FAMEs were quantified with gas chromatography as previously described [20,26].

### 2.4. Starch Content Quantification

For starch analysis, glucose converted from the starch was quantified using anthrone-sulphuric acid colorimetry as previously described [27,28]. Briefly, the cell pellets from 1.0 mL of cell culture were re-suspended in 1.0 mL 80% ethanol (*v*/*v*) and then sonicated three times with a 5-sec interval cycle. The suspension was subsequently autoclaved at 120 °C for 15 min. Enzymic hydrolysis of the starch was initiated by amyloglucosidase (1.5 U) and incubated for 2 h at 55 °C. After the addition of 150 μL anthrone chemical agent, the mixture was subsequently incubated for 10 min at 100 °C, then measured at 621 nm using spectrophotometry (SpectraMax M2e, Molecular Device, Sunnyvale, CA, USA). 

### 2.5. AGPase Activity Assays

The total proteins were extracted from the *Chlamydomonas* cells following the method described previously [25,29]. The activity of AGPase was determined photometrically using the AGPase analysis kit (Solarbio, Beijing, China) according to the manufacturer’s instructions. Protein concentrations were quantified using a BCA protein assay kit (Takara Biomedical Technology (Beijing) Co., Beijing, China). 

### 2.6. Determination of ROS Levels

The cell-permeable fluorogenic probe 2′,7′-dichlorodihydrofluorescein diacetate (DCFH-DA, Beyotime, Shanghai, China) was used to determine the ROS levels as previously described [25,30]. Briefly, the *Chlamydomonas* cells (20 × 10^6^ cells mL^−1^) were collected by centrifugation, washed once with 1 × PBS buffer (0.01 M), and resuspended in 500 μL 1 × PBS buffer containing 0.5 μL DCFH-DA. The blank control was made with 500 μL 1 × PBS buffer containing 0.5 μL DCFH-DA. After incubation at 37 °C in the dark for 30 min with gentle shaking, the samples were washed once and resuspended in 500 μL 1 × PBS buffer. The resuspended culture was examined using an automatic microplate reader SpectraMax M2e (Molecular Device, Sunnyvale, CA, USA) for DCF fluorescence detection (excitation at 488 nm and emission 525 nm). 

### 2.7. Biomass Determination

Briefly, the cell culture (5.0 mL) was filtered using preweighted 47 mm diameter Whatman GF/C filters and dried at 80 °C for 24 h in an incubator. The biomass of the algal cells was then gravimetrically measured as previously described [25,30].

### 2.8. Analysis of Photosynthetic Parameters

The chlorophyll fluorescence of the intact cells was measured using pulse-amplitude-modulated fluorometry (Water-PAM, WALZ, Heinz Walz GmbH, Pfullingen, Germany). Briefly, the cells were dark-adapted for 10 min before applying a saturating pulse (4000 μmol m^−2^ s^−1^, 0.8 s). The maximal quantum yield of PS II (*F_v_/F_m_*) and the effective capability of PS II (*ΔF/F_m_*′) were as we previously described [20,30]. 

### 2.9. Statistical Analysis

All the experiments were carried out in three biological replicates with three technical replicates. The statistical analysis was performed using Tukey’s honestly significant difference (HSD) test or the Student’s *t*-test using SPSS 19.0. 

## 3. Results

### 3.1. Oil Content Increased in FAX1-FAX2-ABCA2 OE under N Starvation

**Figure 1:**
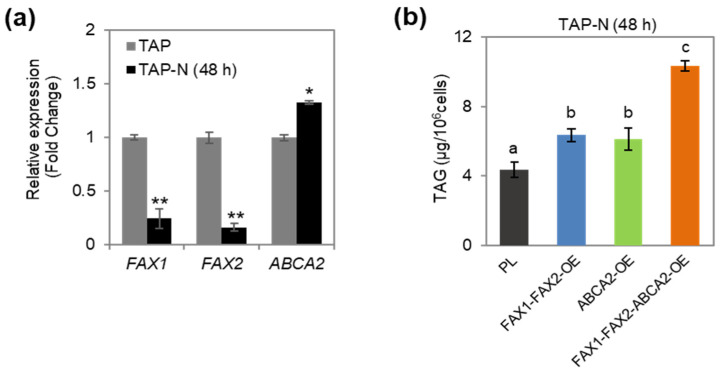
Oil content analysis of the cells under nitrogen-depleted conditions (TAP-N) for 48 h. (**a**) Quantitative RT-PCR (RT-qPCR) analysis of transcriptional responses of *FAX1*, *FAX2*, and *ABCA2* to different nitrogen status in *Chlamydomonas* wild-type strain. The RT-qPCR results were normalized using the housekeeping gene *RACK1* as the internal standard, and the fold changes of the relative expression levels were determined relative to normal conditions (TAP). Error bars represent standard errors based on four biological replicates. Statistical analysis was conducted using Student’s *t* test (* *p* ≤ 0.05; ** *p* ≤ 0.01). (**b**) TAG content in the transformants under TAP-N for 48 h. Data are based on Chen et al. 2022 [20]. PL, untransformed parental line. Statistically significant difference by Tukey’s HSD test was indicated by distinct letters.

To study the function of lipid transporters (FAX1, FAX2, and ABCA2) in lipid metabolism under stress conditions, the expression levels of these genes in the wild-type strain under N starvation were firstly analyzed by quantitative RT-PCR. The results revealed that *FAX1* and *FAX2* were downregulated by 75% and 83% under N starvation (TAP-N) compared with normal cultivation (TAP), respectively (Figure 1a). However, the expression of *ABCA2* was upregulated by 32% under TAP-N compared with TAP (Figure 1a), which is consistent with previous reports [15]. These results indicated that the expressions of these lipid transporters were differently regulated under N starvation. Therefore, we examined whether overexpression of *FAX1* and *FAX2* combined with *ABCA2* may overcome the restrictions of transcriptional regulations, thus further boosting lipid accumulation during N starvation. The single, dual, and triple over-expressors of lipid transporters *ABCA2*-OE, *FAX1-FAX2*-OE, and *FAX1-FAX2-ABCA2*-OE were generated in our previous studies [15,20], and the increased expression levels of *FAX1*, *FAX2*, and *ABCA2* in these transformants under N starvation were determined (Figure S1). We found that the TAG content increased (>40%) in *FAX1-FAX2*-OE and *ABCA2*-OE after N starvation for 48 h compared with the untransformed parental line (PL) (Figure 1b). Strikingly, *FAX1-FAX2-ABCA2*-OE accumulated more TAG than did *FAX1-FAX2*-OE or *ABCA2*-OE. Moreover, the TAG content in *FAX1-FAX2-ABCA2*-OE was 2.4-fold higher than in PL (Figure 1b). TAG yield was also improved as a fraction of dry biomass (Figure S2a). The large increase in oils in *FAX1-FAX2-ABCA2*-OE suggested that the cellular TAG pool is further augmented by co-expression of *FAX1*, *FAX2,* and *ABCA2* during N starvation. Overall, these results indicated that simultaneous overexpressions of these lipid transporters showed a synergetic effect for enhancing oil accumulation under N starvation. 

### 3.2. Major Membrane Lipid Levels Were Enhanced in FAX1-FAX2-ABCA2-OE

**Figure 2:**
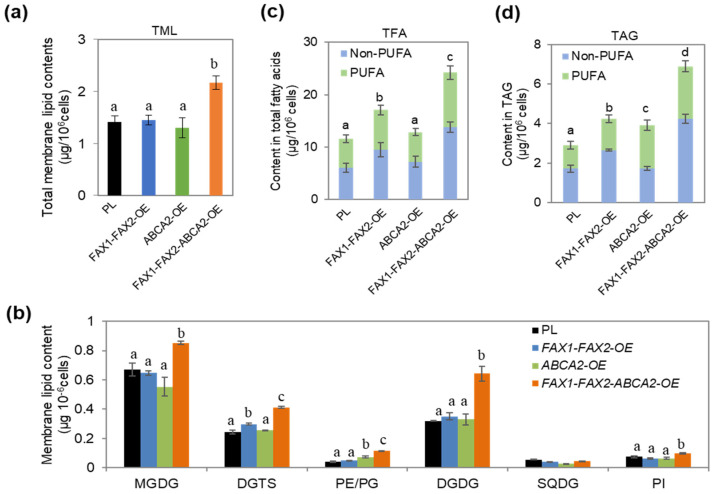
Alterations of the membrane glycerolipid levels and fatty acid compositions under TAP−N for 48 h. (**a**) Total membrane lipid contents. (**b**) Profile of individual membrane component content. (**c**,**d**) PUFA and non−PUFA content in TFA and TAG, respectively. PUFA, polyunsaturated fatty acids; non−PUFA, monounsaturated and saturated fatty acids; TFA, total fatty acids; TAG, triacylglycerols; PI, phosphatidylinositol; DGDG, digalactosyldiacylglycerol; DGTS, diacylglycerol-N,N,N-trimethylhomoserine; SQDG, sulfoquinovosyldiacylglycerol; PE, phosphatidylethanolamine; MGDG, monogalactosyldiacylglycerol; PG, phosphatidylglycerol. Error bars represent standard errors calculated from four biological replicates. Statistically significant difference by Tukey’s HSD test was indicated by distinct letters.

The content of major membrane glycerolipids under N starvation was also analyzed to find out whether the increase in TAG in the transformants causes changes in polar lipids. The results showed that there are no changes in the total membrane lipid (TML) content in *FAX1-FAX2*-OE or *ABCA2*-OE, while the TML increased by 54% in *FAX1-FAX2-ABCA2*-OE compared with the parental line (PL) (Figure 2a). TML also increased as a fraction of dry biomass (Figure S2b). The analysis of membrane glycerolipid composition revealed that the contents of all the major membrane glycerolipid compositions increased in *FAX1-FAX2-ABCA2*-OE compared with individual lipid transporter-OE and PL (Figure 2b). For example, the amount of the major thylakoid membrane lipids (MGDG and DGDG) including the major extraplastidic lipid DGTS increased prominently in *FAX1-FAX2-ABCA2*-OE, whereas no changes in MGDG and DGDG were evident in either *FAX1-FAX2*-OE or *ABCA2*-OE compared with PL (Figure 2b). In *FAX1-FAX2*-OE, the DGTS content was also higher than PL, while there were no changes in DGTS in *ABCA2*-OE (Figure 2b). These results indicate that the main membrane lipids (DGDG, MGDG, and DGTS) also increased in *FAX1-FAX2-ABCA2*-OE. Therefore, the higher TAG did not result in a decrease in membrane lipid content in *FAX1-FAX2-ABCA2*-OE under N starvation. Moreover, we found that total FA (TFA) content significantly increased in *FAX1-FAX2-ABCA2*-OE under N starvation (Figures S2c and S3). Noticeably, *FAX1-FAX2-ABCA2*-OE showed more PUFA and non-PUFA (saturated and monounsaturated) than did any of the control strains. For example, the PUFA content in TFA and TAG increased significantly by 86% and 124% compared with PL, respectively (Figure 2c,d). 

### 3.3. Expression Levels of Key Genes in Lipid Metabolism in FAX1-FAX2-ABCA2-OE

**Figure 3:**
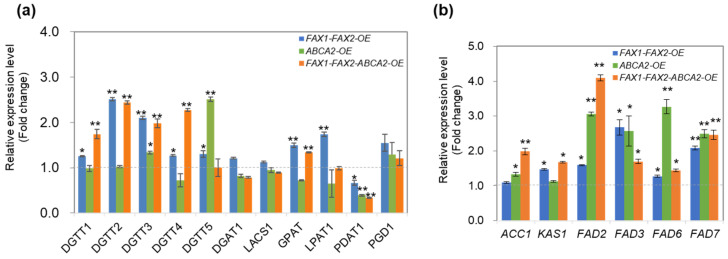
The transcriptional levels of key genes involved in TAG (**a**), and FA biosynthesis (**b**) under TAP-N for 48 h analyzed by RT-qPCR. ACC1, acetyl-CoA carboxylase; KAS1, 3-ketoacyl-CoA-synthase; DGAT, diacylglycerol acyltransferases; DGTT, type II diacylglycerol acyltransferases; FAD, fatty acids desaturases; GPAT, Glycerol-3-phosphate acyltransferase; LPAT1, lysophosphatidic acid acyltransferase 1; LACS1, long-chain acyl-CoA synthetase 1; PGD1, plastid galactoglycerolipid degradation 1; PDAT1, phospholipid:diacylglycerolacyltransferase 1; PUFA, polyunsaturated fatty acids. The RT-qPCR results were normalized using the housekeeping gene *RACK1* as the internal standard, and the fold changes of the relative expression levels were determined relative to the parental line (PL). Error bars represent standard errors based on four biological replicates. Statistical analysis was conducted using the Student’s *t* test (* *p* ≤ 0.05; ** *p* ≤ 0.01).

To better understand the molecular mechanism of enhanced TAG accumulation in the transformants, we performed a transcriptional expression analysis of several key genes in TAG, FA biosynthesis, and membrane lipid remodeling. *FAX1-FAX2-ABCA2*-OE showed different expression patterns between *DGAT* (diacylglycerol acyltransferases) and *PDAT* (phospholipid:diacylglycerolacyltransferase) (Figure 3a). Among the five annotated type II DGAT genes, the transcript levels of the four genes (*DGTT1*–*DGTT4*) were significantly upregulated in *FAX1-FAX2-ABCA2*-OE compared with PL, except that *DGTT5* remained unchanged (Figure 3a). The expression of *GPAT* (glycerol-3-phosphate acyltransferase) was also upregulated, while that of *DGAT1* (type-I DGAT), *LACS1* (long-chain acyl-CoA synthetase), and *LPAT1* were unaltered or slightly downregulated in *FAX1-FAX2-ABCA2*-OE compared with PL. Strikingly, in *FAX1-FAX2-ABCA2*-OE, the genes involved in FA biosynthesis and desaturation, such as *ACC1* (acetyl-CoA carboxylase) and *KAS1* (3-ketoacyl-CoA-synthase) and FA desaturases (*FAD2*, *FAD3*, *FAD6* and *FAD7*), were significantly upregulated (Figure 3b). These results suggest that *FAX1-FAX2-ABCA2*-OE showed a higher expressions of type-II DGATs and FA biosynthesis genes than did PL, which probably caused massive oil accumulation in *FAX1-FAX2-ABCA2*-OE under N starvation. In addition, the expression level of *PDAT1* was downregulated and *PGD1* (MGDG-specific lipase) expression was unaltered in *FAX1-FAX2-ABCA2*-OE (Figure 3a). These results also indicate that the enhanced TAG and TFA are mainly due to an increase in lipid biosynthesis rather than to a recycling of membrane lipid content under N starvation. The downregulated expression of *PDAT1* in lipid transporter-OE might facilitate maintaining the structure and stability of thylakoid membrane lipids under N starvation. 

### 3.4. Starch Content Increases in FAX1-FAX2-ABCA2 OE under N Starvation

**Figure 4:**
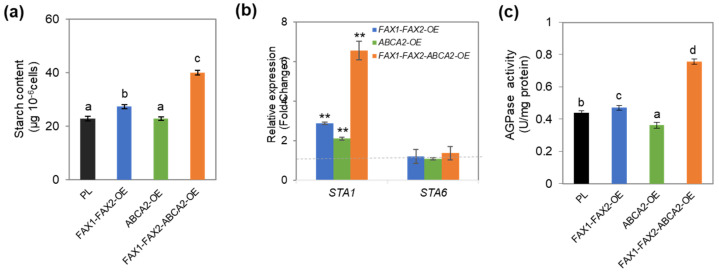
Starch content and expression levels of key genes in starch biosynthesis under TAP−N for 48 h. (**a**) Starch content. (**b**) Transcriptional expression levels of key genes in starch biosynthesis. STA1, the large subunit of ADP−glucose pyrophosphorylase (AGPase); STA6, the small subunit of AGPase. The RT−qPCR results were normalized using the housekeeping gene *RACK1* as the internal standard, and the fold changes of the relative expression levels were determined relative to the parental line (PL). Error bars represent standard errors based on four biological replicates. (**c**) The analysis of AGPase activity. Asterisks and distinct letters labelled indicate significant differences by Student’s *t* test (** *p* ≤ 0.01) and Tukey’s HSD test, respectively.

Starch synthesis involves the same carbon precursor pool as with lipids [31]. To determine whether the TAG increase was caused by the inhibition of starch biosynthesis, the starch content was analyzed. In *FAX1-FAX2*-OE and *ABCA2*-OE, the starch content increased only marginally or remained unaltered compared with the parental line (PL) under N starvation (Figure 4a and Figure S2d). The most significant increase in starch content was found in *FAX1-FAX2-ABCA2*-OE (Figure 4a and Figure S2d). *FAX1-FAX2-ABCA2*-OE showed 1.8-fold more starch than did PL under N starvation (Figure 4a). Interestingly, the starch content also increased by 53% more than in PL under the normal condition (Figure S4). The transcriptional expression and enzymatic activity of ADP-glucose pyrophosphorylase (AGPase) were analyzed, since AGPase catalyzes the initial committed step of starch biosynthesis. The expression level of *STA1* (the large subunit of AGPase) was considerably elevated in the lipid transporter-OE, while that of *STA6* (AGPase small subunit) was unaltered compared with PL (Figure 4b). Notably, the transcriptional expression of *STA1* increased 6.5-fold in *FAX1-FAX2-ABCA2*-OE compared with PL. Furthermore, AGPase activity was also enhanced in *FAX1-FAX2*-OE and *FAX1-FAX2-ABCA2*-OE, whereas that in *ABCA2-OE* was marginally lower than in PL (Figure 4c). The minor differences between AGPase expression and activity are consistent with the unaltered starch content in *ABCA2-OE* compared with PL under N starvation (Figure 4). Overall, these results suggest that co-expression of these FA transporters also has a synergetic effect on increasing starch synthesis, which is due to the upregulated expression of *STA1* and increased enzymatic activity of AGPase. Moreover, these results also indicate that the enhanced TAG accumulation in *FAX1-FAX2-ABCA2*-OE does not affect starch biosynthesis. 

### 3.5. Physiological Characteristics of FAX1-FAX2-ABCA2 OE under N Starvation

**Figure 5:**
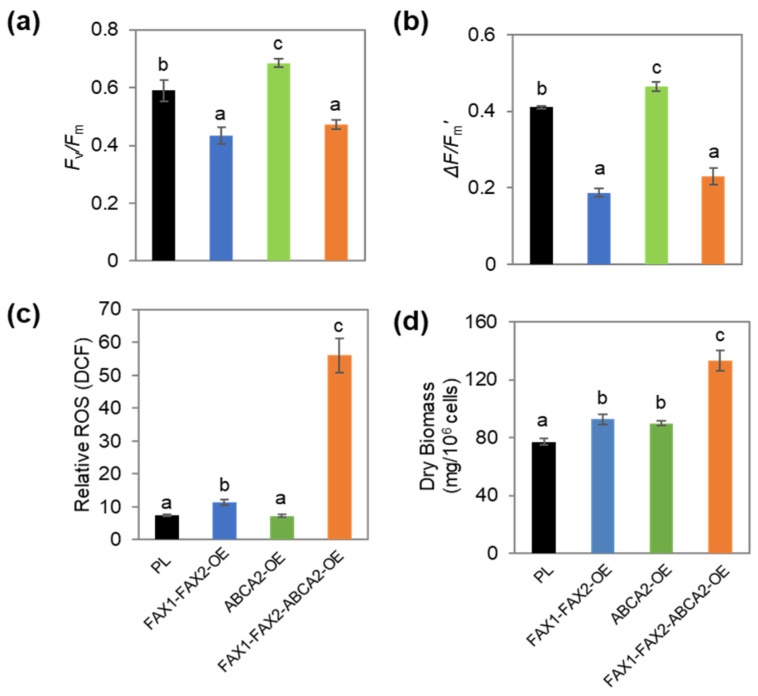
Phenotypic changes in transformants under N starvation conditions. (**a**) Maximum quantum efficiency of PS II (*F_v_/F_m_*). (**b**) Effective capability of PS II (*ΔF/F_m_′*). (**c**) Reactive oxygen species (ROS) content. (**d**) Biomass content. Cells were harvested after N starvation for 48 h. Error bars represent standard errors based on four biological replicates. Statistically significant difference by Tukey’s HSD test was indicated by distinct letters.

To examine whether the remodeling of membrane lipids causes changes in photosynthetic activity in the cells when *FAX1*, *FAX2*, and *ABCA2* were co-expressed, we performed chlorophyll fluorescence measurements. *FAX1-FAX2-ABCA2*-OE and *FAX1-FAX2*-OE showed significantly lower maximum quantum efficiency (*F_v_/F_m_*) and effective photosynthesis capability of PS II (*ΔF/F_m_′*) compared with PL, whereas *ABCA2*-OE exhibited slightly higher *F_v_/F_m_* and *ΔF/F_m_′* (Figure 5a,b). In *FAX1-FAX2-ABCA2*-OE, the reactive oxygen species (ROS) level was 7.4-fold higher than in PL (Figure 5c), whereas the ROS level increased only marginally or remained unaltered in *FAX1-FAX2*-OE and *ABCA2*-OE. These results suggest that the photosynthetic activity in *FAX1-FAX2-ABCA2*-OE was significantly affected by the remodeling of the membrane lipids and the oxidative stress damage of the ROS. In addition, the results showed that the cellular dry biomass increased significantly in all lipid transporter-OE (Figure 5d). Particularly, the dry biomass of *FAX1-FAX2-ABCA2*-OE was higher than in all the control strains. For example, the dry biomass increased by 73% in *FAX1-FAX2-ABCA2*-OE compared with PL (Figure 5d). These results reveal that the increased biomass could be due to the enhancement of lipids and starch content in *FAX1-FAX2-ABCA2*-OE. 

## 4. Discussion

Microalgae accumulate abundant triacylglycerols (TAG) under stress conditions, especially during N starvation. Lipid transporters synergistically contribute to TAG accumulation under normal conditions in plants including microalgae [20,32]; however, their effects on lipid metabolism and related mechanisms are unknown under stress conditions. Here, we tested the effect of combined expression of the lipid transporters (*FAXs* and *ABCA2*) on lipid metabolism during N starvation in the green microalga *Chlamydomonas*. Moreover, transcriptional and physiological analyses were performed to reveal the potential mechanisms of triple over-expressors of lipid transporters when responding to N starvation. Our results suggest that *FAX1-FAX2-ABCA2* overexpression not only has a synergetic effect on enhancing cellular lipid accumulation but also improves starch and biomass content under N starvation. We provide a combined strategy to increase the production of energy-rich carbon reserves in the eukaryotic unicellular microalga *Chlamydomonas* UVM4 strain. Even though the UVM4 strain is derived from the arginine prototrophic strain *cw15-302*, it has been used for many microalgal biology and biotechnology studies, such as valuable bioproduct production, protein localization, and recombined protein secretion [12,21]. Therefore, the strategy developed here might also be applicable to other microalgae. 

**Figure 6:**
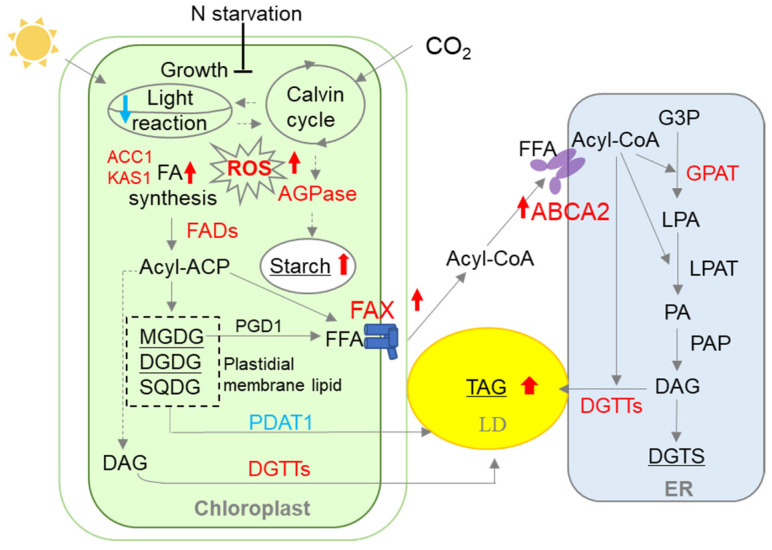
Hypothetical model explaining adjustment of lipid and starch metabolism with increased expressions of *FAXs* and *ABCA2* in *Chlamydomonas* under N starvation conditions. ABCA2, ATP-binding cassette transporter subfamily A2; FAX, fatty acids exporters; ACC1, acetyl-CoA carboxylase; DGTTs, type II diacylglycerol acyltransferases; DGDG, digalactosyldiacylglycerol; G3P, glycerol3-phosphate; ACP, acyl carrier protein; DAG, diacylglycerol; LPAT, lysophosphatidic acid acyltransferase; MGDG, monogalactosyldiacylglycerol; PA, phosphatidicacid; LPA, lysophosphatidicacid; PAP, phosphatidic acid phosphatase; PGD1, plastid galactoglycerolipid degradation 1; DGTS, diacylglyceryltrimethylhomoserine; LD, Lipid droplet; KAS1, 3-ketoacyl-CoA-synthase; SQDG, sulfoquinovosyldiacylglycerol; PDAT1, phospholipid:diacylglycerolacyltransferase 1; ER, endoplasmic reticulum; FFA, free fatty acids; TAG, triacylglycerol. The increased metabolites are underlined. The upregulated and downregulated key genes involved in lipid and starch biosynthesis are colored in red and blue, respectively.

In plants, such as *Arabidopsis thaliana*, overexpression of *AtFAX1* (FA transporter) or *AtABCA9* (ABC transporter) increased TAG accumulation [33,34]. The homologous genes of *AtFAX1* were also recently identified and characterized in microalgae, such as *Chlamydomonas* and *Cyanidioschyzon merolae* [16,35]. Similarly, overexpression of *CrFAX1* and *CmFAX1* increased the TAG accumulation in *Chlamydomonas* and *Cyanidioschyzon merolae*, respectively [16,35]. Up to now, only one homologous gene of *AtABCA9* (*CrABCA2*) was identified and characterized in microalgae. It was reported that CrABCA2 facilitates TAG accumulation in *Chlamydomonas* under N starvation [15]. Even though *FAX1*, *FAX2*, and *ABCA2* are important for oil accumulation, their expression levels were differentially expressed under N starvation (Figure 1a). *ABCA2*-OE cannot accumulate as much oil content as *FAX1-FAX2-ABCA2*-OE (Figure 1b), which is probably due to downregulation of endogenous *FAX1* and *FAX2* during N starvation (Figure 1a). Similarly, the increase in TAG content in *FAX1-FAX2*-OE is less than *FAX1-FAX2-ABCA2*-OE during N starvation (Figure 1b), which might be due to lack of sufficient ABCA2 that transports substrates for TAG biosynthesis to the ER. Therefore, accumulation of TAG content in the single over-expressor might be restricted by the functional diversity or spatiotemporal expression regulation of these lipid transporters. In this study, when *FAX1*, *FAX2*, and *ABCA2* were co-expressed, the triple over-expressor (*FAX1-FAX2-ABCA2*-OE) showed more TAG and membrane lipids than did all the control lines under N starvation (Figure 1b and Figure 2a). The expression levels of type-II DGAT (*DGTT1*–*DGTT4*), ACCase (*ACC1*), FA synthase complex (*KAS1*), and FA desaturases (*FAD2*, *FAD3*, *FAD6*, and *FAD7*) that are involved in TAG and FA synthesis were significantly upregulated in *FAX1-FAX2-ABCA2*-OE. However, the multifunctional galactolipase *PDAT1* was significantly downregulated, and there were no changes in MGDG-specific lipase *PGD1* (Figure 3), which is consistent with increased membrane lipids in *FAX1-FAX2-ABCA2*-OE (Figure 2b). It was reported that galactoglycerolipid pools were the major source of FA esterified in TAG following N deprivation [36]. Overall, the enhanced TAG in *FAX1-FAX2-ABCA2*-OE under N starvation may be caused by the enhanced lipid biosynthesis, or at least may not mainly be due to the recycling of membrane lipid content (Figure 6). However, under normal conditions, lipid biosynthesis and membrane lipid remobilization for TAG biosynthesis were both enhanced when three FA transporters were co-expressed [20]. Therefore, the mechanisms of *FAX1-FAX2-ABCA2*-OE responding to N starvation might be different from those under normal conditions, which will be studied in detail in the future. 

The changes in FA composition, distribution, and transport rate from the chloroplast to the ER may impact photosynthetic activity in microalgae [37]. Therefore, the lower PS II activity in *FAX1-FAX2-ABCA2*-OE might be due to the changed homeostasis of free FA levels (Figure 5a,b). Recently, it was reported that the value of photosynthetic reduction was correlated with N stress–induced TAG accumulation in microalgae [28,38]. For example, the chlorophyll fluorescence parameter *ΔF/F_m_′* was identified as an N stress indicator for TAG production in microalgae [38]. Moreover, it was reported that the decrease in the maximum quantum efficiency of PS II (*F_v_/F_m_*) was positively correlated with the increase in starch content [39]. Therefore, the relative lower *ΔF/F_m_′* and *F_v_/F_m_* are possible reasons for the increased TAG and starch content in *FAX1-FAX2-ABCA2*-OE under N starvation. Under physiological stress, the lower *ΔF/F_m_′* and *F_v_/F_m_* may indicate increasing the availability of reducing equivalents (NADPH) or electrons required for TAG and starch biosynthesis; this will be examined in a future study to decipher the mechanisms involved. Besides TAG and starch, the dry biomass also increased in *FAX1-FAX2-ABCA2*-OE (Figure 5d). These results revealed that carbon precursor fluxes to lipids and starch metabolism may be enhanced in *FAX1-FAX2-ABCA2*-OE under N starvation, and the increased biomass could be due to the enhancement of lipids and starch content. 

In plants and microalgae, the photosynthetic membrane is commonly remobilized to prevent photochemical damage under the changeable environment [40]. For example, the membrane lipids (e.g., MGDG and DGDG) are degraded under adverse conditions in microalgae [5,41]. The fatty acids released from membrane lipids are then used as precursors to synthesize TAG. In *FAX1-FAX2-ABCA2*-OE, the ratio of MGDG to DGDG declined by 37% in comparison with PL (Figure 2b). Under stress conditions, the MGDG/DGDG ratio changes to adapt to stresses and maintain the thylakoid membrane structure [42]. Therefore, the imbalance in the MGDG/DGDG ratio in *FAX1-FAX2-ABCA2*-OE might be associated with membrane lipid remodeling and cause decreased photosynthetic activity (e.g., lower *F_v_/F_m_* and *ΔF/F_m_′*) (Figure 5a,b). The *Chlamydomonas pgd1* mutant, defected in MGDG degradation, exhibited increased ROS production during N deprivation [43]. In this study, co-expression of *FAX1-FAX2-ABCA2* resulted in a reduced ability to manage photosynthetic membrane components (Figure 2b), probably causing ROS over-accumulation (Figure 5c). ROS are key secondary messengers and mediators in microalgae which are involved in metabolite accumulation [44]—for example, over-accumulation of ROS accompanied by an increase in carbohydrate and lipid accumulation in *Chlamydomonas* under a high salinity stress condition [45]. Therefore, the higher intracellular ROS content in *FAX1-FAX2-ABCA2*-OE is also one possible reason for the increased accumulation of TAG and starch (Figure 6). 

Lipid and starch synthesis share some common precursors. Blocking starch biosynthesis has been proposed to increase lipid content in microalgae [31,46]. However, the starch-deficient mutant (*ST68*) of *Chlorella sorokiniana* showed no increase in storage lipids compared to the wild type, which indicates that complicated regulatory steps exist to control carbon re-allocation for lipid and starch biosynthesis in microalgae [6]. In this study, *FAX1-FAX2-ABCA2*-OE showed 1.8-fold more starch than the untransformed parental line (PL), while that in *FAX1-FAX2*-OE and *ABCA2*-OE increased marginally or remained unaltered compared with PL under N starvation (Figure 4a). These results indicated that co-expression of *FAX1*, *FAX2*, and *ABCA2* also has a synergetic effect on enhancing starch synthesis under N starvation (Figure 6). Therefore, the enhanced TAG accumulation in *FAX1-FAX2-ABCA2*-OE does not affect starch biosynthesis. Among all the major enzymes involved in starch biosynthesis, AGPase catalyzes the initial committed step of starch biosynthesis in plants including microalgae [31,47]. AGPase activity is positively correlated with starch content [31]. In plants, AGPase is composed of two large subunits and two small subunits. However, only one large subunit (STA1) and one small subunit (STA6) were functionally characterized in *Chlamydomonas* [48]. The expression level of *STA1* was considerably elevated in *FAX1-FAX2-ABCA2*-OE, while that of *STA6* was unaltered compared with PL (Figure 4b). AGP activity was higher in *FAX1-FAX2-ABCA2*-OE than in the control (Figure 4c). These results indicate that the upregulated expression of *STA1* is the major contributor to the increase in AGP activity and results in a greater starch accumulation in *FAX1-FAX2-ABCA2*-OE compared with PL (Figure 6). Taken together, these results indicate that co-expression of lipid transporters may impact carbon assimilation or fixation ability. Therefore, isotope labeling will be performed in a future study to trace the flow of carbon fluxes to reveal the mechanisms involved. Overall, the genetically synergetic regulation of lipid transporters is a promising strategy for the production of energy-rich carbon reserves in the green microalga *Chlamydomonas* and is a strategy that could be applied to other oleaginous microalgae. 

## 5. Conclusions

In this study, the triple over-expressors of lipid transporters (FAX1, FAX2, and ABCA2) showed increased lipid and starch accumulation as well as improved biomass content under N starvation. These results were further supported by the transcriptional analysis of key genes in lipid and starch metabolism. *FAX1-FAX2-ABCA2*-OE also showed altered photosynthesis activity and increased ROS levels during N deprivation. The strategy developed here could be applied to other microalgae to produce FA-derived energy-rich or value-added compounds.

## Data Availability

The data presented in this study are available within the article and its Supplementary Materials.

## Supplementary Materials

The following supporting information can be downloaded at: https://www.mdpi.com/article/10.3390/metabo13010115/s1, Figure S1: Quantitative RT-PCR analysis (RT-qPCR) analysis of the expression of FAX1, FAX2, and ABCA2 in the transformants under N starvation for 48 h. PL, parental line (UVM4). Error bars represent standard errors based on four biological replicates. Asterisks indicate statistically significant changes compared PL by paired-sample Student’s *t* test (** *p* ≤ 0.01), Figure S2: The contents of TAG, total membrane lipid, total fatty acids and starch under N starvation for 48 h in the transformants analyzed based on dry weight biomass (DW). (a) TAG contents. (b) Total membrane lipid contents. (c) Total fatty acids. (d) Starch contents. Error bars represent standard errors based on four biological replicates. The distinct letters labelled indicate the statistically significant difference by Tukey’s HSD test, Figure S3: The profile of individual fatty acid content of total fatty acids (TFA) under N starvation for 48 h in the transformants were analyzed. Error bars represent standard errors based on three biological replicates. Data are based on Chen et al. (2022). The distinct letters labelled indicate the statistically significant difference by Tukey’s HSD test, Figure S4: The starch content under normal culture condition (before nitrogen starvation). Error bars represent standard errors based on three biological replicates with three technical replicates each. The distinct letters labelled indicate the statistically significant difference by Tukey’s HSD test, Table S1: All primer sequences used for RT-qPCR in this study.
