# Supplementary material for: Co-Expression of Lipid Transporters Simultaneously Enhances Oil and Starch Accumulation in the Green Microalga Chlamydomonas reinhardtii under Nitrogen Starvation
Source: Metabolites. 2023 Jan 10;13(1):115. doi: 10.3390/metabo13010115 (PMC9866645; doi:10.3390/metabo13010115)
Supplement: Supplementary file 1 [file metabolites-13-00115-s001.zip › metabolites-2091618-supplementary.pdf]

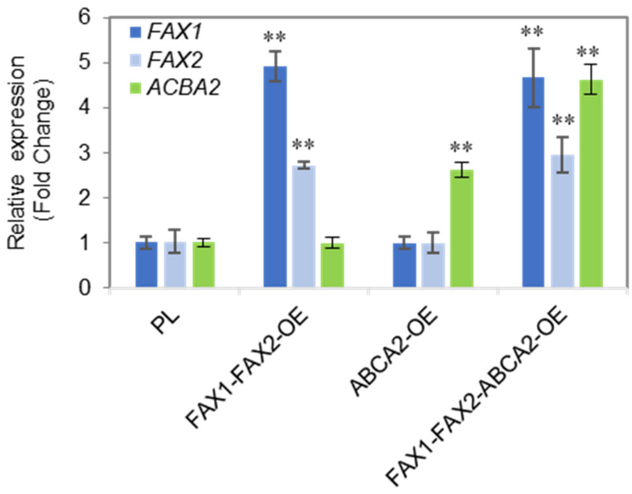

Figure S1. Quantitative RT-PCR analysis (RT-qPCR) analysis of the expression of *FAX1*, *FAX2*, and *ABCA2* in the transformants under N starvation for 48 h. PL, parental line (UVM4). Error bars represent standard errors based on four biological replicates. Asterisks indicate statistically significant changes compared PL by paired-sample Student's t test (**$P \leq 0.01$).

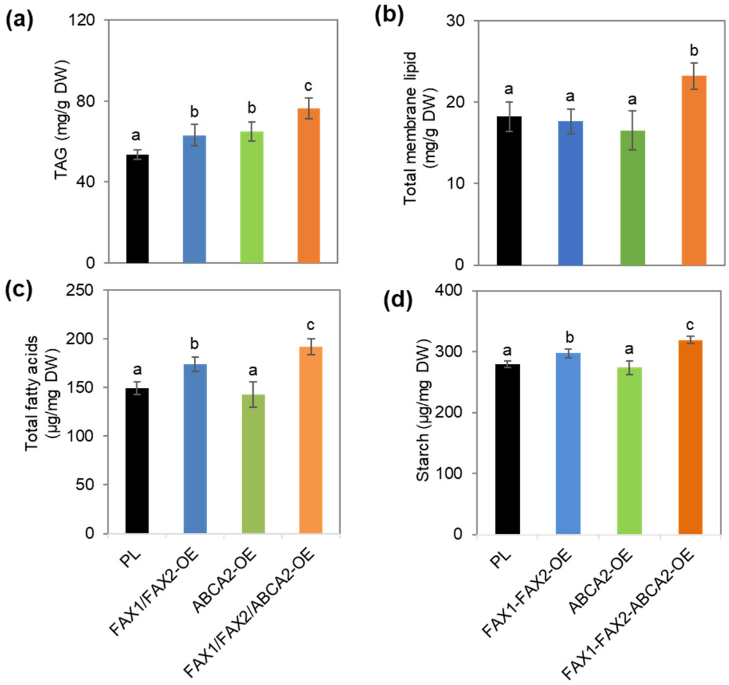

Figure S2. The contents of TAG, total membrane lipid, total fatty acids and starch under N starvation for 48 h in the transformants analyzed based on dry weight biomass (DW). (a) TAG contents. (b) Total membrane lipid contents. (c) Total fatty acids. (d) Starch contents. Error bars represent standard errors based on four biological replicates. The distinct letters labelled indicate the statistically significant difference by Tukey's HSD test.

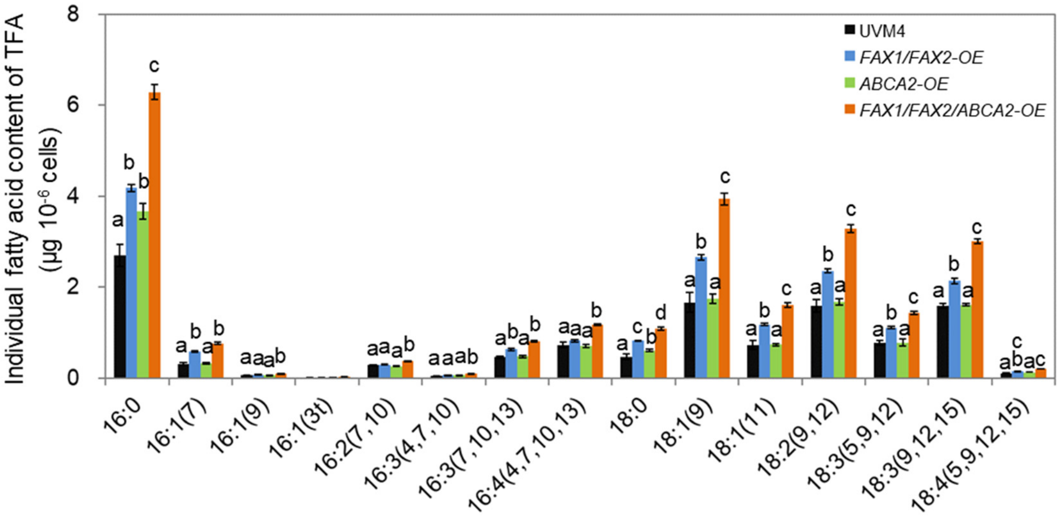

Figure S3. The profile of individual fatty acid content of total fatty acids (TFA) under N starvation for 48 h in the transformants were analyzed. Error bars represent standard errors based on three biological replicates. Data are based on Chen et al. (2022). The distinct letters labelled indicate the statistically significant difference by Tukey's HSD test.

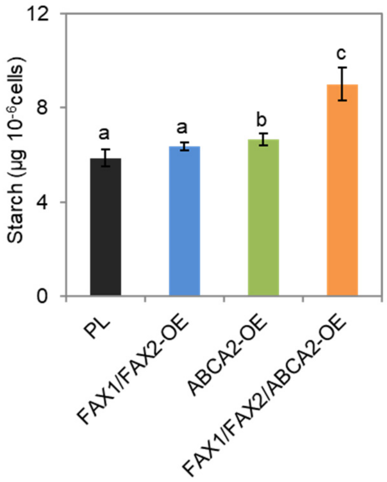

Figure S4. The starch content under normal culture condition (before nitrogen starvation). Error bars represent standard errors based on three biological replicates with three technical replicates each. The distinct letters labelled indicate the statistically significant difference by Tukey's HSD test.

Table S1. All primer sequences used for RT-qPCR in this study.

Target gene	Primer name	Sequence (5′-3′)
RACK1	RACK1-F1	CTTCTCGCCCATGACCAC
	RACK1-R1	CCCACCAGGTTGTTCTTCAG
PDAT1	PDAT1-F	CTGCCTCGCCTCAATGCCTACC
	PDAT1-R	CAGCAGGCCGTTATAGGCGCTG
DGAT1	DGAT1-F	GCTCAACGTGCTAGCAGAGCTGA
	DGAT1-R	CCGTATGGCTGGGAAGTAGACGT
PGD1	PGD1-F	AGCCAGCTATTGTCGCACTT
	PGD1-R	CAAGAAATCCGCTGACATCCGT
ABCA2	ABCA2-qF1	CGTCGCTCAAGGAGTTTGCGCA
	ABCA2-qR	TTGAGCCCCAGCTCCTTCTCCG
FAX1	FAX1-qF	TTCCCTGCTGCAACGATCCCT
	FAX1-qR	GGCTGGCCCTAGATGTGGATGT
FAX2	FAX2-qF	GTCTTCCTGGCCCTAGCGGGT
	FAX2-R-qPCR	CGGCTTGCAGACGGCTTTATGCT
DGTT1	CrDGTT1-F	CGGGCTCTAAAGATGACGCCAACT
	CrDGTT1-R	CGTGCGGGTGGCTCACGAATATAT
DGTT2	CrDGTT2-F	CGCACACCATGTAAATGTTGCGGC
	CrDGTT2-R	TTTGGCAACTCTCAGGTGCTGGAC
DGTT3	CrDGTT3-F	CTGAAGAAGGGCAGTGTGGCGGT
	CrDGTT3-R	CCAGCACTTGGCTCTGACCGAAGT
DGTT4	CrDGTT4-F	GTCAGCGCCATCTACACCTCGGT
	CrDGTT4-R	ACACTGAACTGCACGAACCGCTC
DGTT5	CrDGTT5-F	GCTGAAAGGCCCGAAGGACGAT
	CrDGTT5-R	AGGATCTTGCTGTTGCCCATGTGG
DGAT1	qDGAT-F	GCTCAACGTGCTAGCAGAGCTGA
	qDGAT-R	CCGTATGGCTGGGAAGTAGACGT
LACS1	qLACS1-F	CGTCGGATACTGGCAGGGTG
	qLACS1-R	ATAAGCCGGGACAGGAAGC
GPAT	qGPAT-F	ATCAGCGGTTGTCGAAAACGTCG
	qGPAT-R	GATGTTCCAGTTCCAGGGCTCCG
ACC1	ACC1-F	CCAGCGTCTGCAAGTCGCTC
	ACC1-R	GCTGCCGATACCGCACACGAT
KAS1	KAS1-F	TGGAGAAGGAGCGCTGCTCC
	KAS1-R	CCCTTCTGGACCAGGTTGGACA
LPAT1	CrLPAAT1-F	ACAGGCCACGTGATGATAAACCGT
	CrLPAAT1-R	ATCTTGCAGTCCAGGCTGCGAGT
FAD2	FAD2-F	TCCTACATGCCGCACTATCA
	FAD2-R	TGGTACCAGTACACCTGCTC
FAD3	FAD3-F	TGTTCGAGTCCTACCACCTGCG
	FAD3-R	CACGGATGGCGTTGTACAGCTC
FAD6	FAD6-F	TCTATCAGCCCCTGGTACCTGC
	FAD6-R	GATGTGGCCCACAATGTCCTCG
FAD7	FAD7-F	AAGCTGTACGACCACCTGGAGC
	FAD7-R	GTCGTAGTGCGAGCCCTCCTT
STA1	STA1-F	CCCAGTTCAACTCGACCTCCCTGA
	STA1-R	CCCTGGAACCACTCCTTGTCGGT
STA6	STA6-F	ACATCAGCAAGACGGTGCTGGG
	STA6-R	CCGAGTTGAACTGGGTGAGGCAG
